# A Longitudinal Cone Beam Computed Tomography Study of Mandibular Canal Changes During Growth

**DOI:** 10.1111/ocr.70021

**Published:** 2025-09-04

**Authors:** Stephanie H. Chen, Normand Boucher, Chun‐Hsi Chung, Chenshuang Li

**Affiliations:** ^1^ Department of Orthodontics, School of Dental Medicine University of Pennsylvania Philadelphia Pennsylvania USA

**Keywords:** growth and development, mandibular canal, mental foramen

## Abstract

**Introduction:**

The mandibular canal has been considered a stable anatomic reference structure and continues to be recognised as a primary vertical structure in 2D mandibular superimposition. However, whether the mandibular canal is stable in the transverse dimension is unclear.

**Material and Methods:**

This retrospective longitudinal study utilised cone beam computed tomography (CBCT) images of 17 subjects (9.15 ± 1.79 years old at T1 and 11.47 ± 1.82 years old at T2) who did not have orthodontic or orthopaedic intervention. Mandibular superimposition in 3D of T1 and T2 images was performed, and a series of coronal slices was selected antero‐posteriorly. On each coronal slice, the width and height of the mandibular canals, the distance between left and right canals, as well as the distance between each mandibular canal and the buccal, lingual and inferior surface of the mandible were evaluated. The yearly change of each parameter was calculated.

**Results:**

The vertical and horizontal dimensions of the canal were relatively stable in the posterior region of the mandible; both dimensions increased in the anterior region. In addition, a lateral shift of the mandibular canal was observed, but not in a bodily movement fashion nor a straight V‐shape pattern. The mandibular canal location related to the mandibular body also constantly changed during mandibular growth.

**Conclusion:**

In the period of mixed dentition to early permanent dentition, the dimensions and lateral location of the mandibular canal demonstrated significant developmental changes.

## 1 Introduction

The mandibular canal courses internally within the mandible from the mandibular foramen posteriorly to the mental foramen anteriorly, carrying the inferior alveolar nerve and vasculature. The contour of the mandibular canal was identified in Bjork's preliminary mandibular superimposition method as a stable anatomical reference structure as early as 1963, and remained one of five cortical structures useful for quantifying mandibular growth per Bjork and Skieller's study in 1983. The inferior alveolar nerve canal continues to this day to be recognised as a primary vertical structure in 2D mandibular superimposition by the American Board of Orthodontics [1, 2].

These important classical growth studies were conducted using serial lateral cephalograms from a two‐dimensional lateral perspective. The increasing use of cone beam computed tomography (CBCT) imaging in orthodontic practice has provided an opportunity to extend foundational growth principles from two (2D) to three dimensions (3D) and potentially provide better information for clinical diagnosis and care [3]. Pre‐ and post‐treatment image superimposition is an important strategy to assess craniofacial growth and development as well as orthodontic and orthopaedic treatment outcomes. Whether previously defined stable landmarks on 2D images can still be applied in 3D is a relevant and underexplored topic in craniofacial development.

Prior 3D studies on changes of the mandibular canal during growth are limited and not consistent in their findings. When performing 3D mandibular superimposition with CBCT images for growing patients, Ruellas et al. [4] found that the Bjork landmarks consisting of the cortex of the symphysis, mandibular canal and third molar crypts failed to work in most cases, indicating that the Bjork structures exhibit growth changes not previously appreciable in 2D images. In another 3D study of longitudinal CT scans for Apert syndrome patients, Krarup et al. found that the symphysis and mandibular canal were indeed relatively stable from a lateral perspective, consistent with the findings of Bjork and Skieller [5]. From a frontal perspective, however, all cases showed a lateral shift of the canals during growth, which would suggest a lack of stability in the transverse dimension [5]. A lateral shift of the mandibular canals during growth was not observed by Asaria et al., who utilised the CBCT images of 50 adolescent (aged 11–17 years old) subjects who underwent orthodontic treatment. They concluded that the bilateral mental foramina and canals directly below the mandibular first molars were most clinically stable during the 1.5–2 year observation period [6]. Chen et al. studied CBCTs of 20 growing patients (aged 12.5–17.1 years old) taken about 4.6 years apart, and also found that the mental foramina were relatively stable. However, they observed that mandibular foramina and starting points of the mandibular canal changed significantly in transverse and sagittal dimensions [7].

The large discrepancy among studies regarding the positional changes of the mandibular canal might originate from the large age range of subjects involved in each study, the influence of orthodontic treatment, and changes in the landmarks used for measurement. Thus, by utilising the CBCT images of children who did not receive orthodontic or orthopaedic treatment, the current retrospective longitudinal study intended to understand the detailed pattern and extent of mandibular canal changes, which could further our understanding of mandibular growth and development during the period of late mixed dentition.

## 2 Materials and Methods

### 2.1 Dataset Screening

This retrospective longitudinal study was approved by the Institutional Review Board of the (Protocol #844038 and date of approval: 16 September, 2020). The database of study samples utilised in the current study is the result of data collection that began over 10 years ago from various private practices [8, 9]. The subjects in the sample pool had CBCT taken as part of phase I pre‐orthodontic treatment records (T1, mixed dentition), but did not end up undergoing treatment at that time. A few years later, these individuals returned to the same clinic for follow‐up, at which time another CBCT was taken as part of pre‐orthodontic treatment records (T2, permanent dentition). The CBCT images were all taken at 120 kVp and 5 mA, with a volume size of 16X 13 cm^2^, a voxel size of 0.3 mm, and an exposure time of 3.7 s. The following exclusion criteria were applied to screen CBCT images: The subject had a history of craniofacial syndromes, trauma, or large facial asymmetry; the subject had an impacted mandibular tooth (except third molars); the subject had grossly unidentifiable mandibular canals at either time point. After screening, the final study data set consisted of images from 17 children (7 females, 10 males).

### 2.2 CBCT Measurements

**FIGURE 1:**
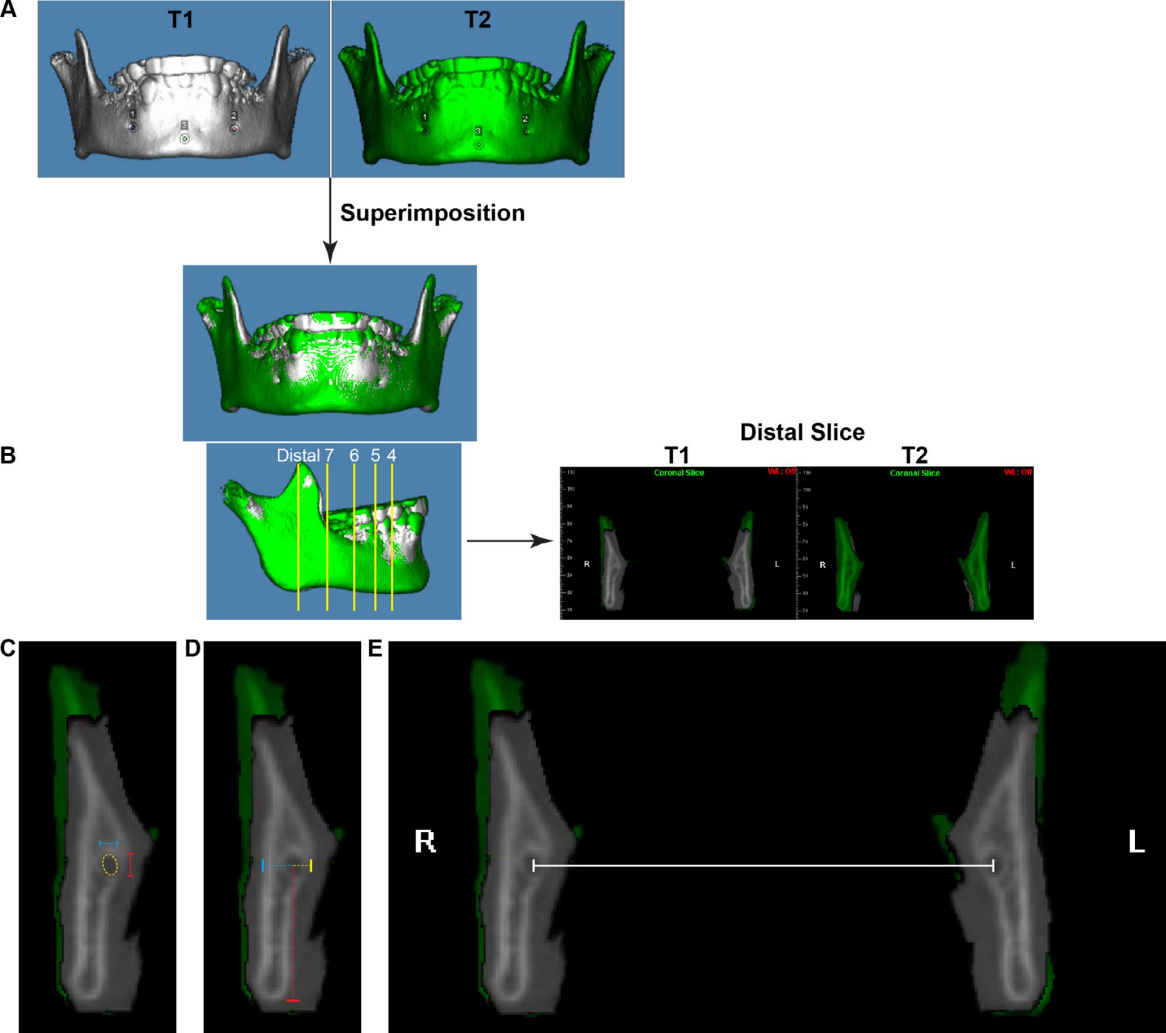
Demonstration of measurement workflow. (A) The 3D images of the mandible from T1 (white) and T2 (green) were oriented based on the mandibular plane and were superimposed using the mandibular symphysis and chin. (B) Five coronal slices were selected on the T2 mandibular body from anterior to posterior: ‘4’—distal to the right first premolar near the mental foramen, ‘5’—distal to the right second premolar, ‘6’—distal to the right first molar, ‘7’—distal to the right second molar and ‘distal’—anterior to the lingula at first full circumferential cortication of the right canal. The measurements were performed at both T1 and T2 images on the same coronal slice. (C, D) At each section, the height (C, red solid line) and width (C, blue solid line) of each canal, as well as distances from each canal centre to the buccal (D, blue dotted line), lingual (D, yellow dotted line) and inferior surfaces (D, red dotted line) of the mandible were measured. (E) The measurement of the transverse distance between right and left canals (inter‐canal distance).

CBCT images were analysed using Dolphin 3D imaging software Dolphin Imaging (version 11.95 Premium, Chatsworth, CA). Mandibles were segmented from the full‐volume CBCT images and oriented using the mandibular plane. The 3D superimposition of T1 and T2 mandibular images was performed as previously described [10] (Figure 1A). Superimposition ensured that all measurements were performed at the same location in the mandible at T1 and T2. Thus, even if tooth location changes occurred due to dentition change (i.e., mesial drift of the mandibular first molar due to loss of E space), the same coronal slice was used for measurements at both time points.

Right and left mandibular canals were identified on coronal slices from the mandibular foramen posteriorly to the mental foramen anteriorly. Five coronal slices were selected on the T2 mandibular body from anterior to posterior (Figure 1B): Distal to the right first premolar near the mental foramen, distal to the right second premolar, distal to the right first molar, distal to the right second molar, and anterior to the lingula at the first full circumferential cortication of the right canal. These sections were defined as 4, 5, 6, 7 and distal, accordingly. The thickness of each coronal slice was set at one voxel.

At each section, the height and width of each canal (Figure 1C), as well as distances from each canal centre to the buccal, lingual and inferior surfaces of the mandible were measured bilaterally (Figure 1D). Additionally, the transverse distance between the right and left canals (inter‐canal distance) was measured at the level of the centre of the mandibular canal (Figure 1E). The inner cortical surface of the canal was used for canal morphology measurements of height and width, while the external cortical surfaces of the mandibular body were used for the distances to the mandibular surfaces.

All the measurements were performed by one examiner. Five samples were randomly selected for another round of measurements by the same examiner 2 weeks later to evaluate the reliability and consistency of the CBCT analysis method.

### 2.3 Statistical Analysis

The intraclass correlation coefficient (ICC) was calculated using IBM SPSS software (Statistical Package for Social Sciences version 26.0, Chicago, IL, USA) to evaluate intra‐examiner reliability. In addition, a paired *t*‐test was performed to evaluate the consistency between the two rounds of measurements. Interpretation was based on guidelines of selecting and reporting ICCs for reliability research [11]: ICC values less than 0.5, between 0.5 and 0.75, between 0.75 and 0.9 and greater than 0.90 were indicative of poor, moderate, good and excellent reliability, respectively.

Power analysis was performed with α = 0.05 and 80% power with an effect size of 1.2. A minimum sample size of 13 was required for comparison between groups, and a minimum sample size of 7 was required for one‐sample Wilcoxon signed‐rank test. The Cohen's *d* of 1.2 was selected to represent a ‘very large’ effect size [12]. Thus, the current database met the minimum sample size requirement but did not allow for comparison between genders. Since no statistically significant difference was detected between the left and right sides, data from both sides were combined for further analysis. As the time interval between T1 and T2 was not identical for all subjects, yearly changes were calculated from linear T2 and T1 differences over time elapsed between the subject's two CBCT images.

Shapiro–Wilk normality test was performed by OriginPro 8 (Origin Lab Corp., Northampton, MA, USA). As some data did not pass the normal distribution test, results are presented with median and 95% confidence intervals overlaying raw data. The data at each location was compared to the theoretical median (0 mm/year) by one sample Wilcoxon signed‐rank test to detect any meaningful yearly changes. In addition, the Friedman test was used for comparison among locations. If a statistically significant difference was detected among locations, Dunn's multiple comparisons test was conducted for the comparison between each two locations.

## 3 Results

### 3.1 Demographic Information

The 17 subjects (7 females, 10 males) were 9.15 ± 1.79 years old at T1 and 11.47 ± 1.82 years old at T2. The time difference between T1 and T2 was 2.32 ± 1.24 years.

Overall, the reliability of the measurement system was high with the ICC ranging between 0.901 and 1.000; however, the canal width measurement had an ICC of 0.738 (Table 1). No statistically significant difference between the two rounds of measurements was detected by the paired‐*t* test.

**TABLE 1:** Interclass correlation coefficients of each measurement parameter.

Parameter	ICC (absolute agreement)	Reliability
Canal width	0.738 [0.626, 0.819]	Moderate
Canal height	0.901 [0.857, 0.932]	Excellent
Inter‐canal distance	1.000 [0.999, 1.000]	Excellent
Buccal‐buccal distance	0.999 [0.998, 0.999]	Excellent
Lingual‐lingual distance	0.999 [0.997, 0.999]	Excellent
Distance to buccal	0.981 [0.972, 0.987]	Excellent
Distance to lingual	0.970 [0.945, 0.982]	Excellent
Distance to inferior border	0.997 [0.995, 0.998]	Excellent

### 3.2 Canal Morphology

**FIGURE 2:**
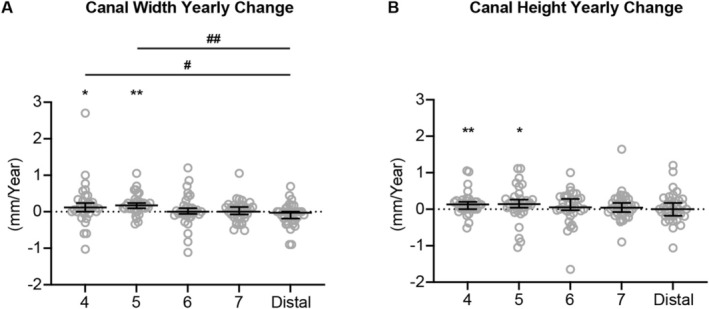
The yearly changes of mandibular canal width (A) and height (B). All the data are presented with the median and 95% confidence intervals overlaying raw data. *Statistically significant compared to the theoretical median (0 mm/year), #Statistically significant when compared between two locations. Single symbol: *P* < 0.05, double symbol: *P* < 0.005.

Annual changes in height and width of the mandibular canal are shown in Figure 2. The vertical and horizontal dimensions of the canal were relatively stable in the posterior portion of the mandible (location 6‐distal), while increases in both canal width and height were detected in the anterior portion (location 4–5).

### 3.3 Canal Direction

**FIGURE 3:**
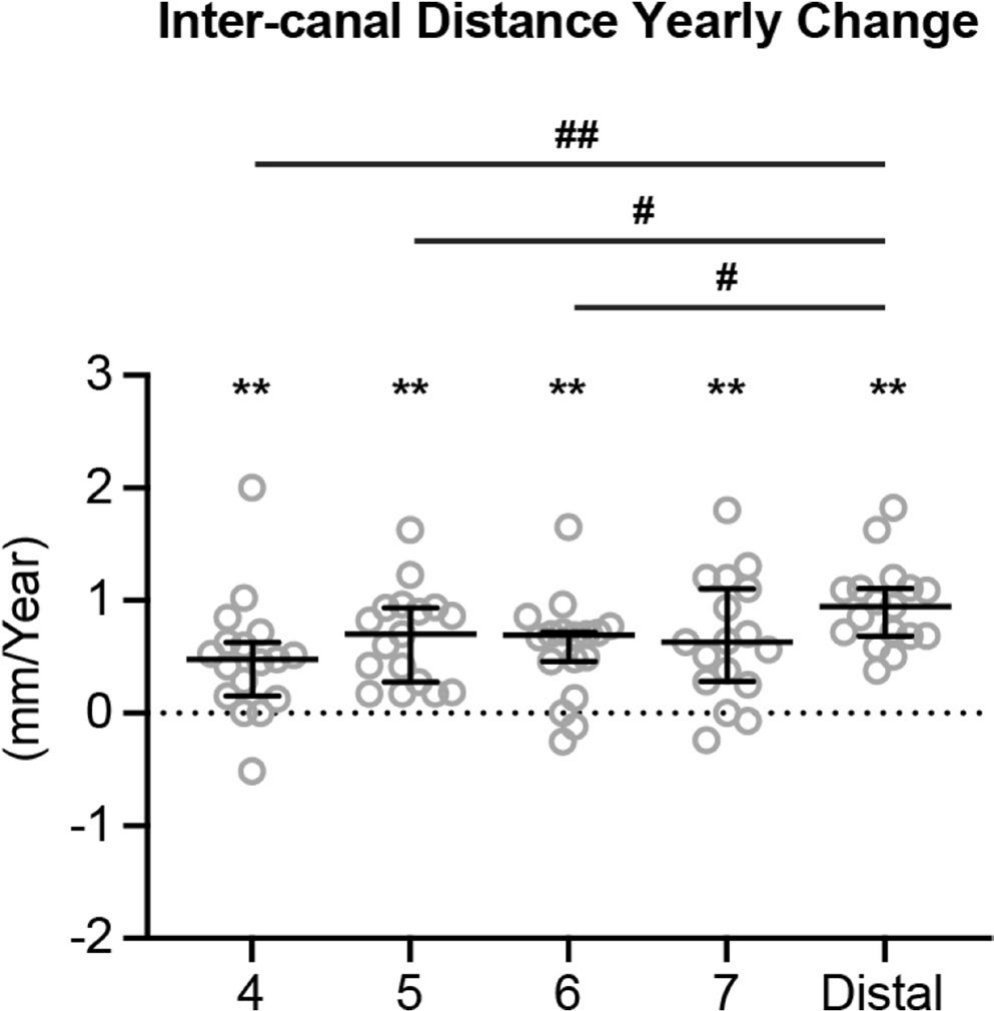
The yearly changes of inter‐mandibular canal distance. All the data are presented with median and 95% confidence intervals overlaying raw data. *Statistically significant compared to the theoretical median (0 mm/year), #Statistically significant when compared between two locations. Single symbol: *P* < 0.05; double symbol: *P* < 0.005.

During this period of mixed dentition growth, the transverse distance between right and left mandibular canals increased in all five locations, indicating a lateral shift of the mandibular canals (Figure 3). When comparing the extent of lateral shift among different locations, the distal site had a greater yearly increase in inter‐canal distance than in the premolar and molar regions and no statistically significant difference was detected among the premolar and molar regions. These findings indicate changes in mandibular canal direction during mandibular growth.

### 3.4 Canal Position

The position of the mandibular canal in the mandibular body was further evaluated using distances between the mandibular canal to the buccal, lingual and inferior external surfaces of the mandible.

**FIGURE 4:**
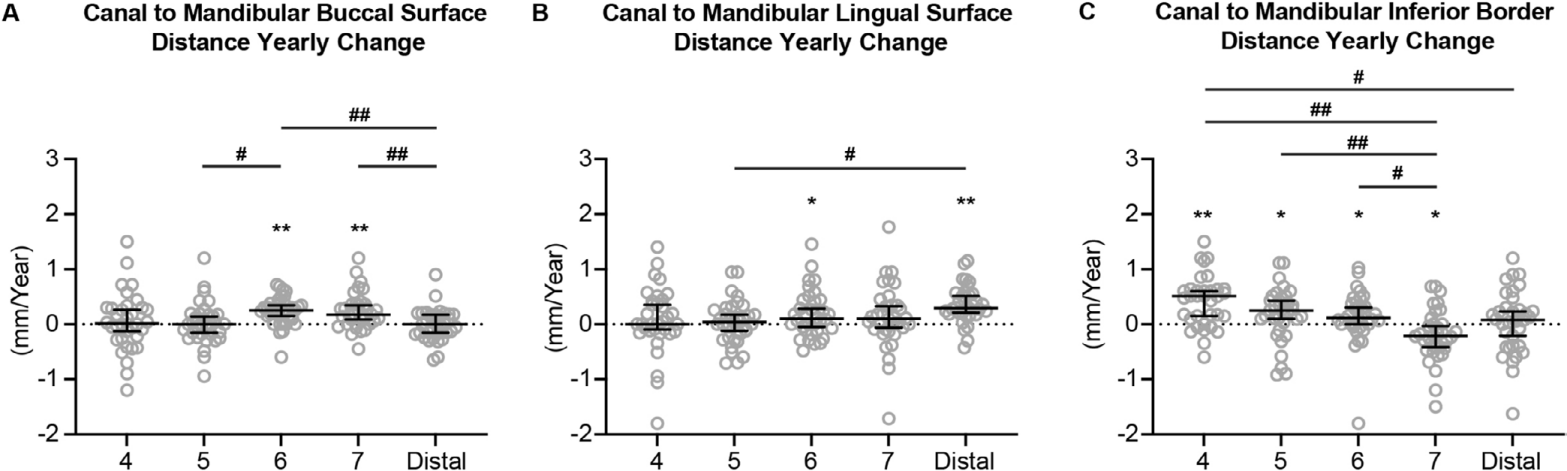
The yearly changes of distance between the mandibular canal and the buccal (A), lingual (B) and inferior (C) surfaces of the mandible. All the data are presented with median and 95% confidence intervals overlaying raw data. *Statistically significant compared to the theoretical median (0 mm/year), #Statistically significant when compared between two locations. Single symbol: *P* < 0.05, double symbol: *P* < 0.005.

With respect to the mandibular buccal surface (Figure 4A), the canal was relatively stable at the premolar and mandibular foramen regions. At the molar region, the distance increased, reflecting a greater horizontal separation between the canal and the buccal surface. The distance from the canal to the mandibular lingual surface also appeared stable around the premolar region (Figure 4B), then increased at the molar and mandibular foramen regions. Vertically, the distance change between the canal and mandibular inferior border increased, then decreased under the dentition moving anterior–posteriorly (Figure 4C). There was no significant distance yearly change detected in the region distal to the dentition. These findings indicate that the mandibular canal position in relation to the mandibular body also experienced constant change in both transverse and vertical directions during mandibular growth.

## 4 Discussion

Based on their vertical stability on 2D lateral cephalograms, mandibular canals are considered stable landmarks during mandibular growth and development, and are routinely used in mandibular superimposition. However, recent studies with 3D imaging have indicated that the mandibular canals may shift laterally with growth, although conclusions from different studies were not consistent and were controversial [5, 6, 7]. In addition, while rare, inferior alveolar nerve damage secondary to orthodontic treatment has been reported [13]. Therefore, fully understanding mandibular canal growth and development is important for orthodontic treatment planning to avoid unnecessary nerve damage and for treatment outcome evaluation by selecting proper reference landmarks. By utilising the longitudinal CBCT images of subjects without orthodontic intervention in a specific period of mandibular growth (from mixed dentition to early permanent dentition), the current study revealed that the mandibular canals do in fact remodel laterally within the mandibular body, but that the rate of remodelling varies with anteroposterior position. In addition, the dimensions of the mandibular canal also undergo constant change, indicating active modelling and remodelling of the mandibular canal during mandibular growth.

Specifically, significant increases in canal width and height were detected in the premolar regions but not in the molar and distal regions (Figure 2). The mandibular premolar region is the location of the mandibular mental foramen. It was reported that the mental foramen is a region that actively remodels in the prenatal stage [14]. In addition, when comparing pre‐ and post‐natal mandibles, the mental foramen changes in a posterior direction as development progresses, which might be due to the development of mandibular dentition [15, 16]. The dimensional changes of the mandibular canal in the premolar region found in this study further demonstrate that the mental foramen undergoes constant modelling and remodelling during the eruption of the permanent dentition. To the best of our knowledge, this finding has not been reported in previous studies.

Along with these dimensional changes, the location of the mental foramen also changed as there was a significantly yearly increase in inter‐canal distance in the premolar region (Figure 3). Thus, in contrast to the studies by Asaria et al. and Chen et al., which reported the mental foramen was clinically stable [6, 7], the current study reported a lateral shift of the mental foramen. It is noted that the age of subjects included in the current study (9.15 ± 1.79 years old at T1 and 11.47 ± 1.82 years old at T2) is different from that in the studies by Asaria et al. and Chen et al. (age 11–17 years old at T1). Thus, differences between current and previous studies regarding the stability of the mandibular mental foramen in the transverse dimension might be due to different stages of mandibular growth.

**FIGURE 5:**
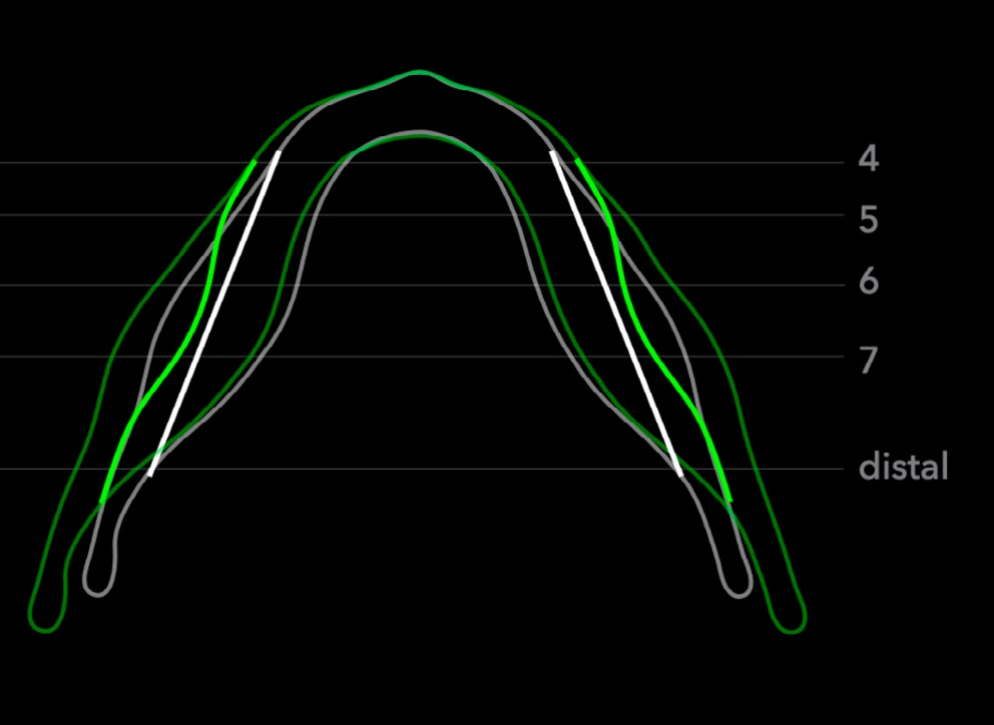
Mandibular canal growth in the transverse dimension. T1 is presented in white and T2 is presented in green. To make visualisation of mandibular canal position changes clearer, the reference base line of the canal position (in white) was set as a straight line. ‘4’—distal to the right first premolar near the mental foramen, ‘5’—distal to the right second premolar, ‘6’—distal to the right first molar, ‘7’—distal to the right second molar and ‘distal’—anterior to the lingula at first full circumferential cortication of the right canal.

In fact, a lateral shift of the mandibular canal was reported by Krarup et al. [5]. However, patients with Apert syndrome were used in the study by Krarup et al., which may not represent normal mandibular canal growth seen in patients without craniofacial syndromes. In addition, perhaps due to the young age of the patients (1 week to 14.5 years old) when taking the CT scans, the mandibular canal was identified in a more posterior location (started at the first permanent molar region) and a description of lateral shifting was not described. In the current study, the rate of lateral inter‐canal modelling varied antero‐posteriorly. There was significantly less inter‐canal distance increase in the first premolar region than in the distal slice (Figure 3). However, the differences did not increase in a gradual pattern antero‐posteriorly, indicating that lateral shifting of the mandibular canal was neither in a bodily movement fashion nor in a straight V‐shape pattern (Figure 5).

Since the mandibular body also experiences transverse growth [10], to further evaluate if the mandibular canal remained in a relatively stable location within the mandibular body, distances between the canal and mandibular buccal or lingual surfaces were measured (Figure 4). Interestingly, different patterns were observed in the distance to the buccal surface and the distance to the lingual surface. For the yearly change of canal to mandibular buccal surface distance, no significant increase in distance was detected except in the molar region (Figure 4A). This increase may be due to the development of the oblique ridge that causes a significant amount of bone deposition on the buccal aspect of the mandible near the molar region [10]. In contrast, for the yearly change of canal to mandibular lingual surface distance, a pattern of gradual increase in distance was observed (Figure 4B). Thus, the mandibular canal location relative to the mandibular body also varied during the mandibular growth.

Lastly, the yearly change of distance between the mandibular canal and the mandibular inferior border demonstrated bone deposition at the anterior and ramus portions of the mandibular border and resorption at the middle portion of the mandibular border. This pattern is consistent with accepted knowledge of mandibular growth based on lateral cephalometric mandibular superimposition and further verified the accuracy of the current measurement system.

There are certain limitations of this study that need to be taken into consideration. Firstly, the findings of the current study may only be applied to a certain period of mandibular growth, i.e., between mixed dentition and early permanent dentition. The mandibular canal may undergo different patterns of remodelling at different developmental periods due to the presence/absence of mandibular primary/permanent tooth buds. Secondly, due to the small sample size, comparison between genders or among different skeletal patterns could not be performed. Previous studies have demonstrated that mandibular sexual dimorphism already exists at 9 years of age [17], and that female and male subjects had different increments and durations of mandibular growth throughout adolescence [10, 17, 18]. In the transverse dimension, median transverse angular growth is greater in males than in females [10]. Additionally, subjects with different skeletal sagittal or vertical patterns also exhibited different amounts of mandibular growth [19, 20, 21]. Thus, whether the pattern and/or extent of mandibular canal remodelling during mandibular growth differs between genders or among different vertical or sagittal skeletal patterns needs further investigation.

## 5 Conclusions

In conclusion, the current study demonstrated that in the period of the mixed dentition to early permanent dentition, both mandibular canal dimension and lateral location demonstrated developmental changes. There is enlargement of the mandibular canal at the premolar region and a lateral shift of the mandibular canal that varies antero‐posteriorly.

## Author Contributions

S.H.C. contributed to the design, data acquisition and interpretation, drafted and critically revised the manuscript. N.B. contributed to data acquisition and interpretation and critically revised the manuscript. C.‐H.C. contributed to critically revising the manuscript. C.L. contributed to the conception, design, data interpretation, drafted and critically revised the manuscript. All authors gave their final approval and agreed to be accountable for all aspects of the work.

## Ethics Statement

The study was conducted in accordance with the Declaration of Helsinki, and approved by the Institutional Review Board of the University of Pennsylvania (Protocol #844038 and date of approval: 16 September, 2020).

## Consent

The authors have nothing to report.

## Conflicts of Interest

The authors declare no conflicts of interest.

## Data Availability

The data that support the findings of this study are available from the corresponding author upon reasonable request.
